# RNA-Seq Analysis of Transcriptome and Glucosinolate Metabolism in Seeds and Sprouts of Broccoli (*Brassica oleracea var. italic*)

**DOI:** 10.1371/journal.pone.0088804

**Published:** 2014-02-27

**Authors:** Jinjun Gao, Xinxin Yu, Fengming Ma, Jing Li

**Affiliations:** 1 College of Life Science, Northeast Agricultural University, Harbin, China; 2 Key Laboratory of Breed Improvement and Physioecology of Cold Region Crops, Northeast Agricultural University, Harbin, China; ISA, Portugal

## Abstract

**Background:**

Broccoli (*Brassica oleracea var. italica*), a member of Cruciferae, is an important vegetable containing high concentration of various nutritive and functional molecules especially the anticarcinogenic glucosinolates. The sprouts of broccoli contain 10–100 times higher level of glucoraphanin, the main contributor of the anticarcinogenesis, than the edible florets. Despite the broccoli sprouts’ functional importance, currently available genetic and genomic tools for their studies are very limited, which greatly restricts the development of this functionally important vegetable.

**Results:**

A total of ∼85 million 251 bp reads were obtained. After *de novo* assembly and searching the assembled transcripts against the *Arabidopsis thaliana* and NCBI nr databases, 19,441 top-hit transcripts were clustered as unigenes with an average length of 2,133 bp. These unigenes were classified according to their putative functional categories. Cluster analysis of total unigenes with similar expression patterns and differentially expressed unigenes among different tissues, as well as transcription factor analysis were performed. We identified 25 putative glucosinolate metabolism genes sharing 62.04–89.72% nucleotide sequence identity with the *Arabidopsis* orthologs. This established a broccoli glucosinolate metabolic pathway with high colinearity to *Arabidopsis*. Many of the biosynthetic and degradation genes showed higher expression after germination than in seeds; especially the expression of the myrosinase *TGG2* was 20–130 times higher. These results along with the previous reports about these genes’ studies in Arabidopsis and the glucosinolate concentration in broccoli sprouts indicate the breakdown products of glucosinolates may play important roles in the stage of broccoli seed germination and sprout development.

**Conclusion:**

Our study provides the largest genetic resource of broccoli to date. These data will pave the way for further studies and genetic engineering of broccoli sprouts and will also provide new insight into the genomic research of this species and its relatives.

## Introduction

Consumption of fruits and vegetables has long been associated with better health and lower incidence of a variety of diseases such as coronary heart disease, cancers, etc [1], [2]. Notably, a diet rich in cruciferous vegetables especially broccoli (*Brassica oleracea var. italica*) has been recognized as an efficient way to reduce the risk of getting many types of cancers. Epidemiological studies prior to 1996 showed an inverse relationship between cancer risk and cruciferous vegetable intake [3], [4]. Some newer studies demonstrate that this inverse relationship is mainly contributed by the breakdown products of glucosinolates [5], [6], [7]. Glucosinolates are a major group of sulfur-rich secondary metabolites specifically in Cruciferae, which are well-known by their breakdown products to display several bioactivities, including plant defense against pathogens and insects as well as anticarcinogenesis in mammals [8]. Based on their precursor amino acids, glucosinolates are divided into three major categories: aliphatic, indolic and aromatic glucosinolates [9], [10]. Among them, the degradation products of aliphatic glucosinolates are considered to have the higher phase 2 detoxication enzyme inducer ability than the other two groups which is effective in blocking chemical carcinogenesis; therefore, they are thought to be the main contributor to protection against carcinogenesis [11].

Although broccoli heads are generally used as the edible part, the sprouts have been suggested to be a better source for health benefits. A study in 1997 reported the sprouts of eight broccoli cultivars have phase 2 enzyme inducer potency (nearly all arose from glucosinolates) 10–100 times greater than that of mature plants [11]. During the first few days of germination, the inducer activity per unit plant weight declined from the maximum point in seeds in an exponential manner. The declining trend flattened after nine days, and finally approached the value in mature broccoli heads after about 15 days [11]. The most valuable information is that in sprouts the aliphatic glucosinolates are dominant, while in adult plant the indolic ones account for the most [12]. The high content of the aliphatic glucosinolates in broccoli sprouts is mainly attributed to glucoraphanin. Its hydrolytic product, sulforaphane, has been well studied with high anticancer activity. It can not only inhibit phase 1 enzymes but also induce phase 2 enzymes [13]. Besides, sulforaphane has an important ability to target the highly aggressive cancer stem cell population, which is responsible for tumor therapeutics and cannot be eliminated by conventional chemo- or radio-therapy [14], [15]. Another interesting fact is that no significant side effects were found in therapy with sulforaphane in the rapeutic concentrations in non-malignant cells or mice [14], [16]. In addition, glucoraphanin has an obvious effect on decreasing oxidative stress, hypertension and inflammation in the cardiovascular system of rats [17]. Based on these promising results, the first prospective clinical studies with cancer patients and sulforaphane-enriched broccoli sprouts have now been initiated in the United States. Therefore, broccoli sprouts should have played a more important role in human health than the mature inflorescences.

The currently available genetic and genomic tools for broccoli research are very limited. While most studies of broccoli focused on physiology, few have been done at the genetic level and functional genomic studies are still in the infancy. Conspicuous effects of ESTs have been reported to develop genetic engineering, including gene family expansion [18], [19], improving breeding programs by SNP and SSR markers [20], [21], facilitating genome annotation [22], and large-scale expression analysis [23], [24]. Currently, only 2,324 broccoli ESTs in the national center for biotechnology information (NCBI) database (http://www.ncbi.nlm.nih.gov/) are generated with the aim to identify gene expression profiles in microspore and floret bud. Most of them have no annotations. Despite of the growing demand and high-yield potential, the average yield of both fresh and processed broccoli has remained virtually unchanged in the United States in recent ten years [25]. Thus, very limited genomic resources of broccoli constitute a key limitation to the development of improved crops. The advent of next generation sequencing technologies has triggered a revolution in biological research, for it is cheaper and more rapid in providing genomic and transcriptomic data [26].

Here we performed a high-throughput Illumina Miseq sequencing to characterize the transcriptomes of five samples, including seeds, cotyledons of 3, 7, 11 day sprouts and euphyllas of 11 day sprouts. Since there is no available reference genome for broccoli, abundant short reads are required in order to perform de novo assembly. From the total of five libraries, we generated 557,094,098 raw reads with an average length of 251 bp containing 139,830,744,098 nucleotide bases. Formal research has suggested that to achieve 99% coverage of an mRNA, at least an 8X sequencing depth is required [27]. For this study, the sequencing depth is 50X, enough to get the maximum coverage. Using a de novo assembly method, 19,441 unigenes are obtained with an average length of 2133 bp. These unigenes are used for subsequent annotation analyses to provide a platform of transcriptome information for genes in broccoli sprouts. In this study, we focused our work on identification of glucosinolate metabolism genes in broccoli seed germination and sprout development. This will pave the way for further genetic engineering to improve this species’ agronomic traits.

## Results and Discussion

### Sequencing and Data Analysis

RNA sequencing of the five samples (seeds, 3 day cotyledons, 7 day cotyledons, 11 day cotyledons and 11 day euphyllas) produced a total of ∼85million 251 bp paired-end reads with an average of 17million reads for each sample. Cleaning and quality checks were performed to the raw data (cf. Materials and Methods). A total of ∼75million trimmed reads were obtained with useful data percentage in five time points ranging from 70.29% to 76.01% and the average length of each read was 207 bp (Table S1). Compared to the reads generated by the formal platforms, the longer length of Illumina Miseq sequencing reads greatly facilitated the accuracy of the subsequent *de novo* assembly. Using single k-mer assembler Velvet (http://www.ebi.ac.uk/~zerbino/velvet), assembly of reads generated 659,752 contigs with mean sizes of 254 bp and N50 of 222 bp (Table 1). The contigs with length more than 500 bp accounted for about 6.19%. Multiple K-mer assembler OASES (http://www.ebi.ac.uk/~zerbino/oases) was applied to produce 122,345 transcripts for 40,081 loci with average length of 1670 bp. Then, all the transcripts were blasted against the *Arabidopsis* database. For those “non-BLASTable” transcripts, we searched them against the NCBI non-redundant (nr) database, using BLASTx program with an E-value threshold of 1E-5. A total of 94,255 (77.04%) transcripts were significantly matched to known genes in *Arabidopsis* and 3,971 (3.25%) transcripts were matched to the nr database. The high percentage of transcripts matched to *Arabidopsis* database is due to the close relation of these two species. For the transcripts representing the same loci, the top hit ones were clustered as unigenes. Finally, 19,441 unigenes were generated, with the average length of 2,133 bp and ranging in size from 200 bp to 20,580 bp (Table 1). The size distribution of contigs, transcripts and unigenes were compiled (Figure S1). The sequencing data has been deposited into NCBI gene expression omnibus (GEO) and the accession number is GSE53298.

**Table 1:** Statistical summary of cDNA sequences of broccoli generated by the Illumina Miseq platform.

	Total length(bp)	Sequence No.	Max Length(bp)	Average Length(bp)	N50	>N50 Reads No.
Contigs	167802146	659752	7524	254	222	205867
Transcripts	204377219	122345	20680	1670	2527	26568
Unigenes	41474146	19441	20680	2133	2631	5234

**Figure 1:**
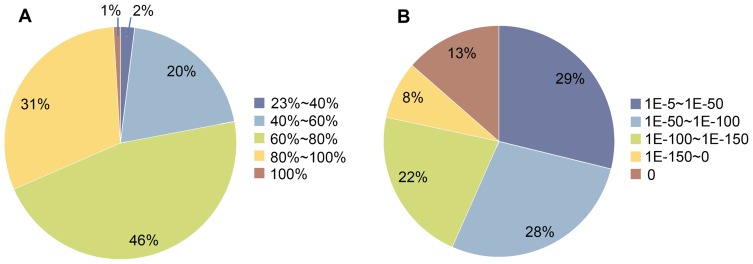
Characterization of broccoli unigenes by searching against public database. A. Identity distribution of unigenes blasted against public databases with E-value cutoff of 1E-5; B. E-value distribution of unigenes blasted against public databases with E-value cutoff of 1E-5.

Variable efficiency of matching to sequences in the databases was found in assembled sequences of different lengths, with longer sequences showing higher match proportions (Figure S2). For sequences longer than 1500 bp, the match efficiency was 98.24%. But for sequences between 200–500 bp and 500–1000 bp in length, it was just 53.40% and 79.17%, respectively. E-value distribution of the top hits in the databases had shown 71.17% of matched sequenced with strong homology (<1.0e-50) (Figure 1). 30.54% of the transcripts had a similarity higher than 80%, while 46.47% showed similarity between 60%–80% in identity distribution pattern. The total 77.01% of the transcripts showing identity higher than 60% along with the high quality e-value distribution supported the reliability of the *de novo* assembly performed in the study.

### Annotation and Classification

**Figure 2:**
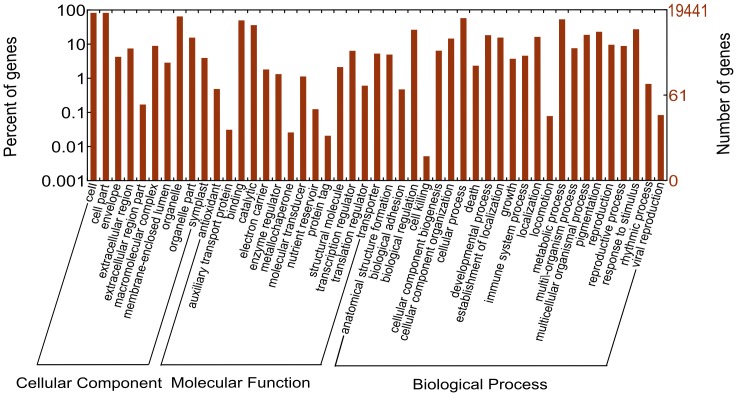
Histogram presentation of Gene Ontology classification of the assembled unigenes.

Since biologists have recognized that there is likely to be a single limited universe of genes and proteins are conserved in most, if not all living cells, the GO (gene ontology) Consortium was created as a joint project of many organism databases to produce a structured, precisely defined, common, controlled vocabulary for describing the functions of genes and gene products in any organisms [28]. To annotate the broccoli transcriptome, GO terms were assigned to broccoli unigenes based on their identity to known protein sequences in the *Arabidopsis* database and nr database. 19,441 unigenes were assigned to 47 functional groups with 134,938 functional terms using GO assignments (Figure 2). For the three main categories of GO classification scheme, the assignments to the “biological process” (61,583, 45.64%) made up the majority, followed by the “cellular component” (53,030, 39.30%) and the “molecular function” (20324, 15.06%). Among these GO groups, the high number of unigenes putatively involved in “cellular process” (11,129) and “metabolic process” (10,230) in the biological process category indicated that the broccoli tissues used in this study were undergoing exquisite metabolic activities, which coincided with the samples’ status. Interestingly, 5,220 unigenes were assigned to “response to stimulus”, showed that during the germination of broccoli seeds and the development of sprouts, there were some protective mechanisms for preparing for potential external and/or internal stresses. Under the category of cellular component, the “cell”, “cell part” and “organelle” were prominent groups. It is noteworthy that the unigenes were not gathered into few groups but were generally expressed. This might be due to the widespread requirements during seedling development.

EggNOG (evolutionary genealogy of genes: Non-supervised Orthologous Groups) is a database providing orthologous groups for 943 Bacteria, 69 Archaea and 121 Eukaryotes [29]. According to the previous report, the proteins could be divided into 25 functional categories [30]. Out of 19,441 unigenes with significant identity with *Arabidopsis* database and nr database in this study, 11,242 could be classified into 24 eggNOG categories with only “Nuclear structure” having no annotated unigenes (Figure S3). The categories “function unknown” (2,088, 18.57%) and “general function prediction only” (2,050, 18.24%) were the two largest functional groups of the 25 eggNOG categories. The high percentage of unigenes classified into “general function prediction only” coincided with the transcriptome studies of other species [31], [32], [33]. But our newly noticed fact that so many unigenes were assigned to unknown functional group might indicate there are some interesting unknown mechanism during germination of broccoli seeds and the development of sprouts. Following the most abundant two groups were “transcription” (929, 8.26%), “replication”, “recombination and repair” (802, 7.13%), “signal transduction mechanisms”(797, 7.09%) and “posttranslational modification, protein turnover, chaperones”(680, 6.05%), whereas the two groups involving “cell motility” and “extracellular structures” consisted of a total of 10 unigenes (0.09%), representing the smallest eggNOG classifications. Noteworthily, 277 unigenes (2.64%) were classified into secondary metabolite biosynthesis group, including glucosinolate biosynthesis in broccoli sprouts.

The KEGG (Kyoto Encyclopedia of Genes and Genomes) is a database linking genomic information with higher order functional information by collecting manually drawn pathway maps representing current knowledge on cellular processes and standardized gene annotations [34]. A total of 9836 genes were classified into six main categories including 38 secondary pathways (Figure S4) in the five tested samples. “Metabolism” is the biggest category (3,624, 36.84%), followed by “human disease” (1,760, 17.89%), “Genetic Information Processing” (1,674, 17.02%), “Organismal Systems” (1,279, 13.00%) and “Cellular Processes” (909, 9.24%), whereas “environmental information processing” (590, 6.00%) containing only 3 sub-units (“membrane transport”, “signal transduction and signaling molecules and interaction”) was the smallest category. These results indicated that the broccoli sprouts were undertaken active metabolic and genetic processes and the functional classification of KEGG provided a valuable resource for investigating specific processes and pathways in broccoli sprouts.

### Gene Expression Pattern

**Figure 3:**
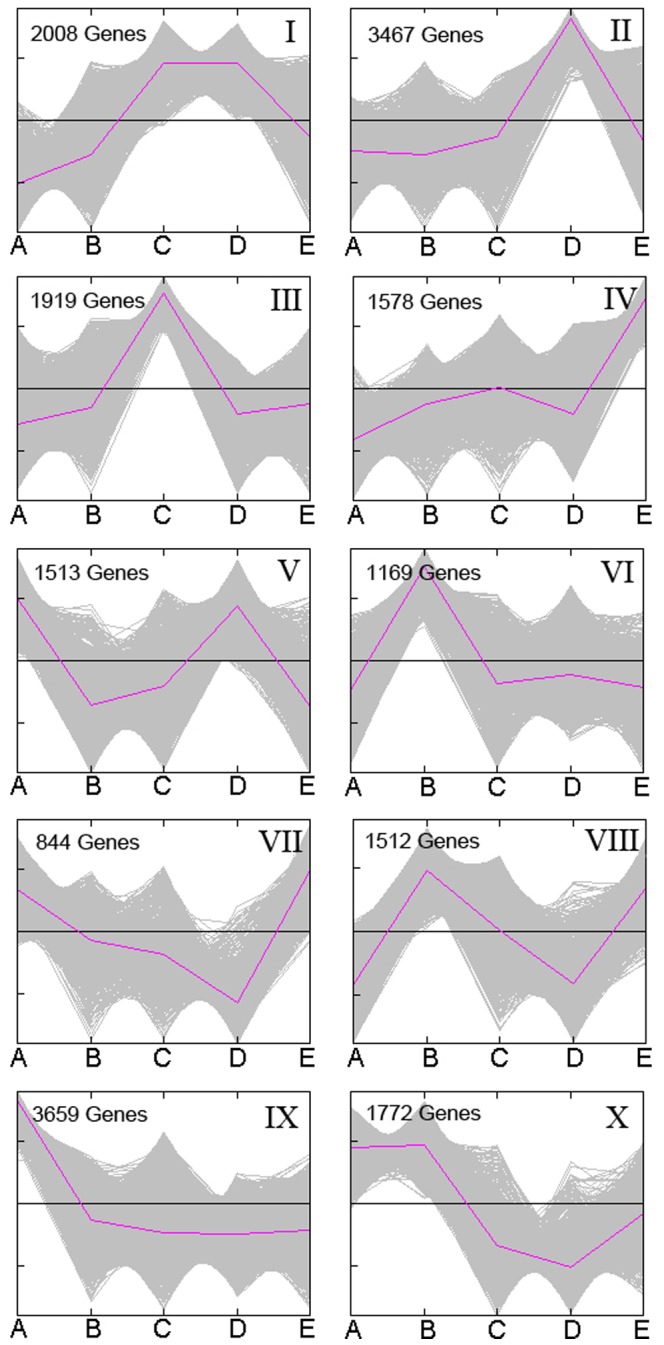
Dynamic expression patterns during broccoli seed germination and sprout development. K-means clustering was performed to identify 10 clusters, each containing various numbers of genes with similar expression pattern during broccoli seed germination and sprout development. The red lines show representative transcriptional regulators. The x-axis represents sequenced samples, and the y-axis represents normalized RNA-seq expression level. A. seeds; B. 3 day cotyledons; C. 7 day cotyledons; D. 11 day cotyledons; E. 11 day euphyllas.

Gene expression patterns can provide important clues as to the roles of unknown genes in biological active processes [35]. While RPKM (reads aligned to gene per kilobase of exon per million mapped reads) was widely used to calculate gene expression values [36], we used a more accurate method called DESeq to estimate gene expression values in this analysis to infer differential expression signals with good statistical power [37]. K-means clustering analysis was performed using the software MeV edition 4.90 (http://www.tm4.prg/mev.html) to group unigenes with similar expression patterns under different time points and resulting in 10 different clusters (Figure 3). The most abundant cluster (IX) contained 3,659 genes with highest expression at the very beginning (i.e. seeds) and then their expression levels were down-regulated all through the development of sprouts, and reached the lowest point at the 11D euphyllas. These genes might greatly and specifically contribute to seed germination. Cluster I, II, III, VI and VIII comprised genes whose expression levels were very low in seeds but peaked at any one of the three points in cotyledons. Also, genes in cluster IV showed the highest expression level at 11D euphyllas with relatively low level at other time points.

**Figure 4:**
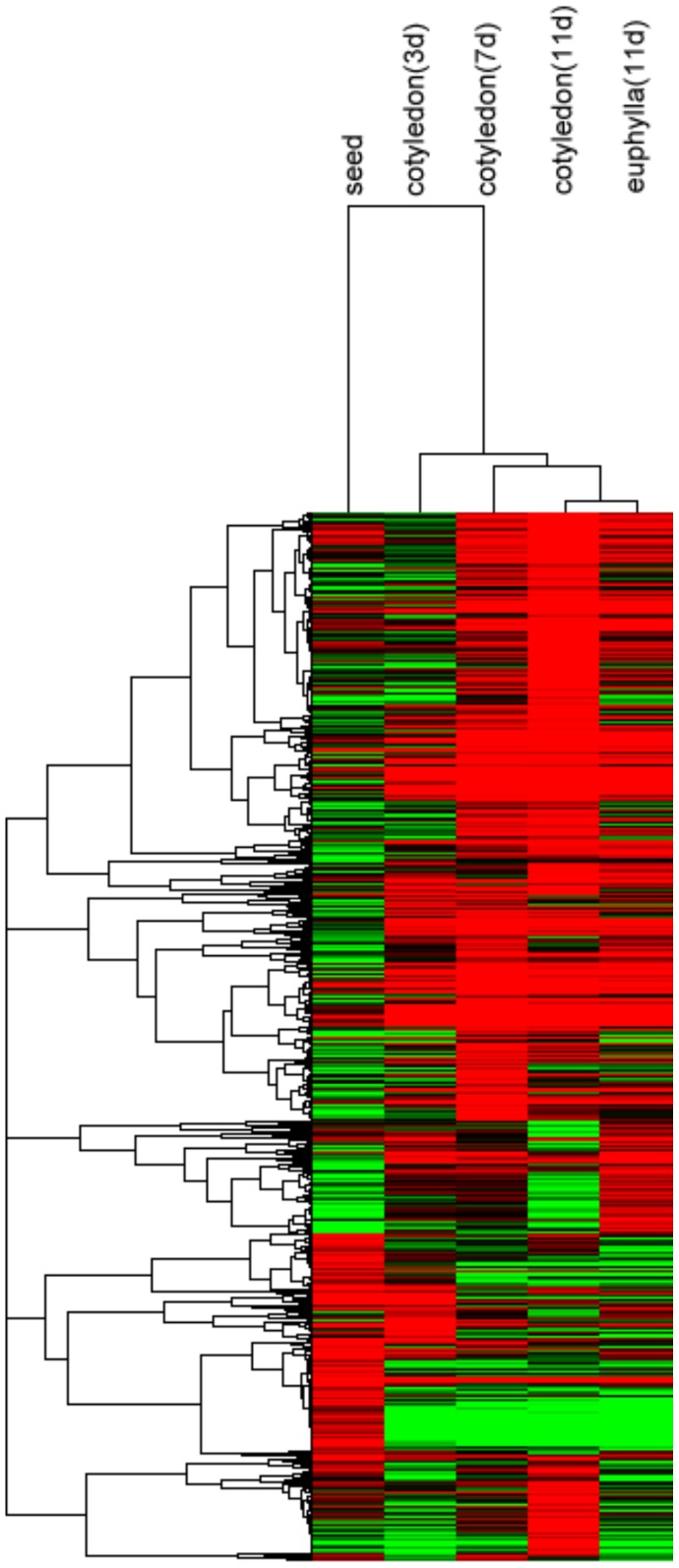
Cluster of differentially expressed genes during broccoli seed germination and sprout development. Expression changes and cluster analysis of 2675 genes that were differentially expressed between any two of the five samples. Each row represents a differentially expressed gene, while each column represents a sample. Changes in expression levels are shown in color scales with saturation at >2.0-fold changes. Green and red color gradients indicate a decrease and increase in transcript abundance, respectively.

In order to identify differentially expressed genes between the five types of samples, we compared them with each other and picked out a total of 2675 genes, which were at least 2-fold up- or down-regulated between two samples with p-value smaller than 0.05. Then, hierarchical cluster was generated to gain a global view of the differential expressed genes (Figure 4). Obviously, the 11 day cotyledons showed closer relationship with the 11 day euphyllas than with the cotyledons of other time points. This indicated the similar function between the initial stage of euphyllas and the late stage of cotyledons. As expected, the three time points of cotyledons were more similar to each other than to seeds. Even though the 7th day is the mid-point of 3rd day and 11th day, the expression profile of 7 day cotyledons was more similar to that of 11 day cotyledons than that of 3 day cotyledons. This fact along with the big difference between seeds and 3 day cotyledons illustrated the 3 day might be the special point in broccoli sprout development.

### Putative Transcription Factors

Transcription factors (TFs) have been considered as one of the most important functional elements regulating gene expression that leads to developmental and other changes. It has been reported that in response to internal or external environment changes, TF genes exhibit more rapid expression changes than the bulk of the regulated genes [38]. Thus, the expression profile of TF genes may in some way reflect the subsequent transcription activities regulated by them. For their important roles, the key putative TFs involved in broccoli seed germination and sprout development were analyzed.

A study in 2003 has revealed that in *Arabidopsis*, most (84%) of TFs could be detected in six day old seedlings [38]. Currently, the Plant Transcription Factor Database (PlnTFDB) contains 2451 and 2162 distinct TF sequences from *Arabidopsis* and *Arabidopsis lyrata,* respectively, arranged in 81 families [39]. In the sequenced broccoli seeds and sprouts, 78 TF families including 1,633 putative TF genes had been identified with the five most expressed TF families being AP2-EREBP, bHLH, MYB, HB and C3H (Table S2). A total of 1,581 of the 1,633 genes accounting for 86.82% were annotated with sequences from the close related species *Arabidopsis* and *Arabidopsis lyrata*.

The biggest TF family in our study of broccoli seeds and sprouts was AP2-EREBP with 109 putative family members being detected. AP2-EREBP family is unique to plants and characterized by a conserved AP2 DNA-binding domain of about 60 amino acids [40]. AP2-EREBP genes have been found to play important roles throughout the life cycle including regulating several developmental processes especially leaf epidermal cell identity and forming part of the mechanisms used to respond to stress [41]. Some members of AP2-EREBP, like *AP2*, control seed mass and seed size in *Arabidopsis* which is very important to extended growth of cotyledons [42], [43]. The significantly large number of AP2-EREBP family members expressed in broccoli seeds and sprouts indicated the important function of these genes in this period as previously reported in other species [41], [42], [43].

The basic helix-loop-helix (bHLH) family members are involved in various process of seedling development such as light signaling, brassinosteroid and abscisic acid signaling, flavonoid biosynthesis, axillary meristem formation, stomatal patterning and trichome differentiation [44]. 99 unigenes identified to have bHLH-like sequences formed the second biggest TF family in the tested samples. Light is one of the most important elements in seed germination and seedling development, a subfamily containing 15 members are involved in light signaling. These bHLH proteins are known as PIF (phytochrome interacting factor) or PIL (phytochrome interacting factor-like) [45], [46], [47], [48]. These proteins play important roles in phytochrome signal transduction by interacting with phytochromes. Our study found that putative orthologs of *bHLH56, PIF1, PIF3, PIF4, PIF5, PIL1, PIF7*, *SPT* were expressed in broccoli seed germination and sprout development, suggesting their similar roles in light signaling during this period. SPATULA (SPT) was found as a leaf size regulator [49]. The SPT ortholog in broccoli sprouts showed highest expression level in the cotyledons of 3 day sprouts, indicating its possible regulation activity on cotyledon size in early broccoli sprouts.

The MYB TFs contain varying numbers of MYB domain repeats to bind DNA. The function of MYB proteins have been well studied in a variety of plant species to be involved in regulatory networks controlling development, metabolism and responses to biotic and abiotic stresses in *Arabidopsis* [50]. In *Arabidopsis* seedlings, MYB115 and MYB118 play important roles in embryogenesis [51]. MYB38 and MYB18 have been proposed to regulate hypocotyls elongation in response to blue [52] and far-red light, respectively [53]. Also, the MYB17 has shown activity in regulating seed germination [54]. Some other MYB proteins are involved in the control of cell wall biosynthesis like MYB58, MYB63, MYB85, MYB68 and MYB46 [55], [56], [57], [58]. In this study, a total of 84 putative MYB genes were detected including those orthologs involved in *Arabidopsis* seedling development. The many putative MYB TF genes expressed in the broccoli seed and sprouts indicated this important family also plays important roles in regulating the biological process during seed germination and sprout development.

Sixty-seven putative NAC TF family members were identified in seed and sprouts of broccoli. The large NAC transcription factor family has been implicated in a variety of plant developmental processes in many species including *Arabidopsis* and soy bean etc [59]. However, the molecular mechanisms of the family members are still unknown even in well studied species. It has been suggested that they have the ability to enable crosstalk between different pathways [60]. Cys2His2 (C2H2)-type zinc finger proteins are a group of widespread eukaryotic TFs. A majority of C2H2 zinc finger proteins are regarded as *trans* regulators of genes playing important roles in development, differentiation and suppression of malignant cell transformation [61]. In the sequenced tissues, 65 putative C2H2 zinc finger genes were identified.

Several other TF families were also found like 61 members in bZIP, 54 members in WRKY, 14 members in ARF, etc. Because of the importance of TFs in regulating the downstream genes in variety of pathways, further investigation of the putative TFs would provide interesting clues to the variety of activities in seed germination and sprout development of broccoli.

### Glucosinolate Metabolic Pathways

**Figure 5:**
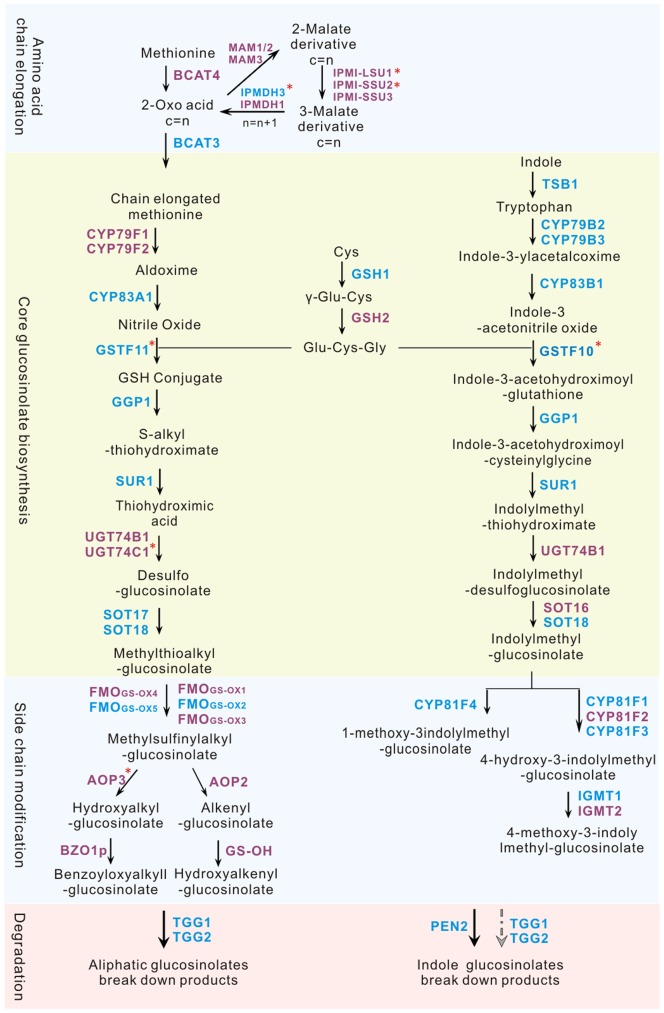
Detected orthologs in the aliphatic and indolic glucosinolate biosynthetic and degradation pathways in broccoli seeds and sprouts. Four stages of the pathways in *Arabidopsis* are showed separately for chain elongation, core structure biosynthesis, side chain modification and degradation. Othologs identified in Broccoli are marked in blue color. Predicted enzymes are marked by *.

The high contents of glucosinolates especially the much higher content of aliphatic glucosinolates in broccoli sprouts compared to mature tissues have attracted attention in past decade [11]. The biological basis of this trait especially whether the glucosinolate metabolic genes in *Arabidopsis* or *Brassica rapa* have the same functions in broccoli sprouts, remains an open question. In this study, a total of 36 unigenes were annotated as putative genes involved in aliphatic and indolic glucosinolate biosynthesis, degradation and regulation. By comparing these unigenes with the CDS of *Arabidopsis* ones and setting the identity cutoff of 60%, we abandoned 11 unigenes and finally got 25 putative broccoli glucosinolates metabolic genes sharing 62.04–89.72% nucleotide sequence identity with the *Arabidopsis* orthologs (Figure 5, Table 2).

**Table 2:** Putative genes involved in glucosinolate metabolic pathways in Broccoli.

Name	*Arabidopsis* orthologs	Basemean of seeds	Basemean of 3 day cotyledons	Basemean of 7 day cotyledons	Basemean of 11 day cotyledons	Basemean of 11 day euphyllas	Identity
**Aliphatic glucosinolates**							
**Glucosinolate synthesis**							
BoIPMDH3**	AT1g31180	9.2	2.03	9.45	2.14	8.09	63.28%
*BoBCAT3*	AT3g49680	274.54	635.68	722.67	482.47	678.31	86.31%
*BoCYP83A1*	AT4g13770	1.42	54.92	922.24	345.23	427.55	88.20%
BoGSTF11**	AT3g03190	16.27	125.1	32.56	2.14	71.64	83.94%
*BoFMO-GSOX2*	AT1g62540	9.91	8.14	534.65	11.79	9.24	76.50%
*BoFMO-GSOX5*	AT1g12140	42.46	15.26	22.06	6.43	18.49	78.61%
*BoSOT17*	AT1g18590	235.63	49.84	306.71	1627.53	116.71	83.00%
**Transcription factor**							
*BoMyb29*	AT5g07690	0	6.1	52.52	0	6.93	83.04%
**Indolic** **glucosinolates**							
**Tryptophan synthesis**							
*BoTSB1*	AT5g54810	421.02	454.63	560.91	263.75	271.55	79.41%
**Glucosinolate synthesis**							
*BoCYP79B2*	AT4g39950	87.03	73.23	829.81	1029.27	145.6	85.41%
*BoCYP79B3*	AT2g22330	0.71	13.22	281.51	306.64	18.49	89.72%
*BoCYP83B1*	AT4g31500	961.62	515.66	1543.03	3386.94	479.55	86.67%
BoGSTF10**	AT2g30870	844.16	1554.1	3612.3	3070.66	1061.95	67.23%
*BoCYP81F1*	AT4G37430	55.19	49.84	3.15	0	0	76.35%
*BoCYP81F3*	AT4G37400	58.73	6.1	52.52	28.95	0	86.83%
*BoCYP81F4*	AT4G37410	378.56	12.2	175.42	8.58	16.18	81.70%
*BoIGMT1*	AT1G21100	89.16	8.14	52.52	700.12	27.73	82.44%
**Transcription factor**							
*BoMyb51*	AT1g18570	90.57	18.31	56.72	131.88	33.51	85.46%
**Common to all glucosinolates**							
*BoSOT18*	AT1g74090	79.25	39.67	53.57	164.04	55.47	64.22%
BoGGP1*	AT4g30530	780.47	474.98	1406.48	7330.34	746.48	81.14%
*BoSUR1*	AT2g20610	179.02	58.99	257.35	536.08	104	75.56%
*BoGSH1/PAD2*	AT4g23100	4680	1962.96	1898.06	5790.72	1453.68	68.61%
**Glucosinolate degradation**							
*BoPEN2*	AT2G44490	247.66	157.65	279.4	2437.01	294.66	70.49%
*BoTGG1*	AT5G26000	55.19	42.72	74.58	111.5	132.89	68.58%
*BoTGG2*	AT5G25980	38.21	1702.59	1590.29	818.06	4958.45	62.04%

The glucosinolate biosynthesis proceeds through three independent stages: chain elongation (for aliphatic glucosinolates), core structure formation and side chain modification (Figure 5) [62]. For the 25 selected putative genes, 7 were uniquely involved in the aliphatic pathway including *BoIPMDH3, BoBCAT3, BoCYP83A1, BoGSTF11, BoSOT17, BoFMO_GS-OX2_ and BoFMO_GS-OX5_*. In the chain elongation stage, two genes (*BoIPMDH3, BoBCAT3*) were detected. *BCAT3* encodes a chloroplast branched-chain amino acid aminotransferase [62] and *IPMDH3* is one of the three isopropylmalate dehydrogenase genes in *Arabidopsis* whose isozyme *IPMDH1* has been characterized as a functional gene involved in aliphatic glucosinolate chain elongation process [63]. Most of the *Arabidopsis* genes involved in biosynthesis of aliphatic glucosinolate core structure have orthologs expressed in the studied tissues except for *CYP79F1, CYP79F2 and UGT74B1, UGT74C1* (Figure 5). It has been reported that in the double-knockout *Arabidopsis* mutant of *CYP79F1* and *CYP79F2*, aliphatic glucosinolate biosynthesis is completely abolished, meaning that *CYP79F1* and *CYP79F2* are necessary for the pathway [64]. The contradiction between the high level of aliphatic glucosinolate content and the missing of both of *CYP79F1* and *CYP79F2* indicates that there may be unknown gene(s) performing the same function in broccoli sprouts. *FMO_GS-OX2_ and FMO_GS-OX5_* are important genes performing S-oxygenation in side chain modification stage in *Arabidopsis* [65], [66], they may have the same function in broccoli. However, orthologs of other genes of this stage expressed in *Arabidopsis* have not been identified in our tissues. Notably, glucoraphanin is one of the products produced by *FMO_GS-OX2_* [66]. The missing of the downstream genes of *FMO_GS-OX2_* may explain the accumulation of glucoraphanin in broccoli sprouts.

In broccoli seeds and sprouts, the indolic glucosinolate biosynthetic pathway showed a high colinearity with *Arabidopsis*. Eight genes involved in the indolic pathway were detected with *BoCYP79B2, BoCYP79B3*, *BoCYP83B1 and BoGSTF10* in core structure formation and *BoCYP81F4, BoCYP81F1, BoCYP81F3* and *BoIGMT1* in side chain modification. The enzyme UGT74B1 transforming the Indolylmethyl-thiohydroximate to the Indolylmethyl-desulfoglucosinolate in the indolic glucosinolate pathway was missing. In *Arabidopsis*, when the indolylmethyl-glucosinolates were formed, there were two ways for them to be modified. Part of them would be transformed to 1-methoxy-3indolylmethyl-glucosinolates by CYP81F4 and the others would be transformed to 4-methoxy-3indolylmethyl-glucosinolates by CYP81F1, CYP81F2 or CYP81F3 and then the IGMT1 or IGMT2 would modify them to 4-methoxy-3-indolylmethyl-glucosinolates [67]. The *CYP81F2* and *IGMT2* were not detected in this study.

Beside these genes uniquely expressed in indolic or aliphatic glucosinolate pathway, the three genes involved in both the two pathways including *GGP1*, *SUR1* and *SOT18* were all identified in the studied tissues too. In *Arabidopsis*, *TSB1* is a tryptophan synthesis gene [68] and *GSH1* is a crucial gene to form GSH, which is considered as the sulfur donor to be conjugated with the activated aldoxime [69], [70]. The orthologs in broccoli with these two genes not involved in glucosinolate biosynthesis directly but having important roles for the forming of glucosinolates were also identified.

Some transcription factors of MYB family are crucial in regulating glucosinolate biosynthesis pathways of *Arabidopsis*, in which MYB28, MYB29 and MYB76 [71], [72], [73] are involved in aliphatic glucosinolates biosynthesis whereas MYB51, MYB34 and MYB 122 [74], [75] could strongly enhance the expression of indolic glucosinolate biosynthesis genes. In broccoli seeds and sprouts, only *BoMYB29* and *BoMYB51* were detected.

In the glucosinolate degradation pathways, the *PEN2*, *TGG1* and *TGG2* were well studied in *Arabidopsis*. *PEN2* was reported to cleave indolic glucosinolates as a myrosinase [76]. TGG1 and TGG2 were two important myrosinases identified long time ago. The double mutant of these two genes showed nearly no aliphatic glucosinolate degradation but only reduced indolic glucosinolate myrosinase activity [77]. In addition, the *tgg1tgg2* double mutant still exhibited a wild type callose response to fungi simulation which required the degradation products of indolic glucosinolates [78]. These facts indicated the TGG1 and TGG2 mainly degrade the aliphatic glucosinolates and had slight effects on indolic ones. In the sequenced tissues, the three myrosinase genes’ orthologs were all identified.

It is interesting to note that the expression levels of many broccoli glucosinolate related genes were expressed higher in sprouts than in seeds. Some previous studies had indicated the glucosinolate concentration decreased exponentially after germination [11], [12]. This contradiction between the decreased concentration level of glucosinolates and the increased level of biosynthesis genes might due to the high consumption of glucosinolates and this dramatic degradation of glucosinolates possibly played an important role in the stage of broccoli seed germination and sprout development.

Besides, the putative genes involved in indolic glucosinolate synthesis have higher expression levels than those involved in aliphatic glucosinolate synthesis in general. This was more obvious in the expression of TFs. The only identified transcription factor *BoMYB29* in aliphatic glucosinolate synthesis had no expression in seeds and 11 day cotyledons; in the other three time points, the expression level was also relatively low (Table 2). The expression values of *BoMYB51* were much higher compared to the expression of *BoMYB29.* Furthermore, we noticed that the expression value of *BoTGG2*, was 45-fold higher in 3 day cotyledons than in seeds (Table 2). The expression value decreased slowly along with the development of sprouts and got to the lowest point in the 11 day cotyledons, which was still about 21-fold higher than that in seeds. Notably, at the time of 11th day, the expression values of *BoTGG2* in the new forming euphyllas astonishingly increased to about 130-fold. While the expression values of the indolic glucosinolate degradation gene *BoPEN2* were relatively low compared to those of *BoTGG2* and not too much different in our sequenced tissues except for the 11 day cotyledons (Table 2). These results demonstrated that in glucosinolate sprouts, the exponentially decreased levels of glucosinolates were mainly due to the degradation of aliphatic glucosinolates especially in the young stage of tissues, while the indolic glucosinolates might be constantly synthesized and stored. The exception was the expression value of *BoPEN2* in late stage cotyledons increased to 10-fold higher than that in seeds. This might indicate the degraded indolic glucosinolates had special roles at the old stage of cotyledons.

Actually, previous studies have reported that the expression of *TGG1*, redundantly functioning with *TGG2* in *Arabidopsis*, is higher in young developing tissues than older tissues [79], [80], [81] and *PEN2* is unlikely to function in glucosinolate turnover during seedling development [82], which are coordinated to our results. Degraded glucosinolates have been proposed to regulate the cellular signaling in response to abiotic stress which is based on the observation that *TGG1* is highly enriched in stomatal guard cells and regulate the stomatal opening or closing by affecting the ABA and MeJA signaling [83], [84]. So the myrosinases’ high level in young seedling may be necessary for proper protection due to lower physical strength barriers. In our study, *BoTGG2* was the predominantly expressed myrosinase rather than *TGG1* in *Arabidopsis*. Considering that *TGG1* and *TGG2* redundantly function in glucosinolate degradation [77], the predominantly functional gene may be *BoTGG2* in broccoli sprouts instead of *BoTGG1.* Also, glucosinolates has been suggested to represent up to 30% of total sulfur content of plant organs and glucosinolate content has been observed to decrease during sulfur deprivation [85]. So we can hypothesis that the enriched degradation products of glucosinolates may contribute greatly to the defense system as a compensation for the lower physical strength barrier in broccoli sprouts and they may also play an important role as potential sulfur donor during broccoli seed germination and sprout development.

### Conclusion

In this study, we performed a transcriptome sequencing of seeds, 3 day, 7 day and 11 day cotyledons and 11day euphyllas to identify the transcripts and quantify their levels of expression in broccoli seed germination and sprout developments. In total, we obtained 19441 unigenes annotated by homologs with sequences in the public databases. 2675 genes differentially expressed between any two of the five samples and 1,633 putative TFs were detected. These genes may be closely related to the seed germination and sprout development in broccoli. Twenty-five unigenes with high identity to *Arabidopsis* glucosinolate metabolic genes have been investigated. This established a high colinearity between *Arabidopsis* and broccoli in glucosinolate metabolic pathways for further studies. In addition, expression analysis of these glucosinolate metabolic genes showed contradiction between the increased expression of the candidate synthesis genes and the previously reported decreased concentration of glucosinolate content after germination. The ortholog of TGG2, which mainly degrade aliphatic glucosinolates in *Arabidopsis*, expressed astonishingly higher after germination. These results indicate the breakdown products of glucosinolates may play important roles in the stage of broccoli seed germination and sprout development. The results here represent the largest genetic resource for broccoli and will provide new insight into the genomic research of this species and its relatives.

## Materials and Methods

### Sample Preparation

**Figure 6:**
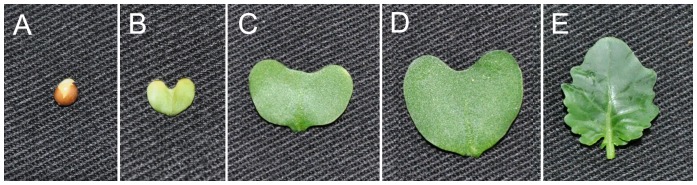
Images of the sampled tissues. A. seed; B. 3 day cotyledon; C. 7 day cotyledon; D. 11 day cotyledon; E. 11 day euphylla.

Seeds of broccoli cultivar “Qingxiu” with wide suitability of temperature and soil were germinated and grown in trays containing a soil mixture (peat: vermiculite, 2∶3, v/v). Plants were adequately watered with Hoagland’s solution and grown in a culture room with the following settings: 24°C, light regime of 16 h light and 8 h dark, 70% relative humidity and a constant illumination of 100 µmol·m^−2^·s^−1^. For the sample of seeds, they were incubated in 5% NaClO with shaking for 8 min and then were washed six times using sterile water with once 30 s. Subsequently, seeds were placed on moist filter paper in petri dishes for one night. Finally, samples of equal weight were harvested for seeds, cotyledons at the 3^rd^ day, 7^th^ day and 11^th^ day and euphyllas at 11^th^ day (Figure 6). To minimize biological variance, each sample was harvested in three independent biological replicates with equal weight and subsequently pooled for sequencing. Samples were immediately frozen in liquid nitrogen and stored at −80°C until RNA was extracted. Total RNA of each sample was isolated using E.Z.N.A. Plant RNA Kit (OMEGA bio-tek, GA) according to the instructions from the manufacturer. RNA quality was characterized on an agarose gels electrophoresis and spectrophotometry. High quality RNA with 28S:18S more than 1.5 and absorbance 260/280 ratios between 1.8 and 2.2 was used for library construction and sequencing.

### cDNA Library Construction and Sequencing

Illumina Miseq library construction was performed according to the manufacturer’s instructions (Illumina, San Diego, CA). Magnetic beads with poly T oligos attached were used for purifying the mRNA from the total RNA. Fragmentation buffer was added to cleave mRNA into short fragments. The fragments were used to synthesize first-strand cDNA using random hexamerprimers, which was transformed into double stranded cDNA with RHase H and DNA polymerase I. A paired-end library was constructed from the cDNA synthesized with Genomic Sample Prep Kit (Illumina). Fragments in desirable lengths were purified with QIAquick PCR (Qiagen) Extraction Kit, end repaired and linked with sequencing adapters (Margulies et al., 2005). AMPureXP beads were used to remove the unsuitable fragments, then the sequencing library was constructed with PCR amplification. After being checked with Pico green staining and fluorospectrophotometry and quantified with Agilent 2100, the multiplexed DNA libraries were mixed by equal volume with normalized 10 nM concentration. The sequencing library was then sequenced with Illumina Miseq platform (Shanghai Personal Biotechnology Cp., Ltd. Shanghai, China).

### Data Filtering and *de novo* Assembly

Raw sequencing reads of five samples were mixed together to perform the following filtration using a stringent process and subsequent *de novo* assembly. The adaptor contamination was removed, the reads were screened from the 3′ to 5′ to trim the bases with a quality score of Q<20 using 5 bp windows and the reads with final length less than 25 bp were removed. De novo transcriptome assembly was performed by Velvet [86] followed by Oases [87] with default settings except for K-mer value to get contigs and transcripts. Velvet was run using single k-mer length of 69 and OASES was then run with the preliminary Velvet assemblies as input. Because the results after merging with multiple k-mers used in OASES program prompted severe assembly redundancy, we use the same single k-mer in OASES program. High quality reads of every sample were remapped to transcripts to get the abundance of transcripts using Bowtie program [88]. Those transcripts with no reads mapped in all five samples were considered error and removed. All the transcripts were searched against Arabidopsis database, for those with no hits were then searched to NCBI non-redundant (nr) database (ftp://ftp.ncbi.nlm.nih.gov/blast/db/) with BLAST program (E-value <1E-5), and the top-hit transcripts were selected as unigenes. For the unigenes failed to be aligned to the databases, the software GetORF [89] was used to predict their open reading frames (ORFs) and ascertain their sequence directions, with default settings except for the parameter “–find” being set 1.

### Gene Annotation and Analysis

To further annotate the unigenes in this study, we used the Blast2GO program [90], [91], [92], [93] to get GO annotation based on GO terms related to the *Arabidopsis* and nr database annotation. EggNOG (evolutionary genealogy of genes: Non-supervised Orthologous Groups) is a database of orthologous groups of genes. To annotate genes with common denominators or functional categories, the unigenes were also aligned to the eggNOG database (http://eggnog.embl.de/version_3.0/). To summarize the pathways information involved in broccoli seeds and sprouts, the KEGG database were used to perform the pathway annotation (http://www.genome.jp/kegg/). To identify putative TFs presented in this research, we searched the unigenes against the complete TF gene sequences of the Plant Transcription Factor Database (http://plntfdb.bio.uni-potsdam.de/) using BLAST program with an E-value cutoff of 1E-5. To identify the putative sequences related to glucosinolate pathways, the unigenes annotated by putative glucosinolate biosynthetic and regulator genes according to previous studies were chosen [9], [66]. Then, CDS of *Arabidopsis* glucosinolate biosynthesis and regulator genes were aligned to broccoli homologs using DNAMAN6.0 (http://www.lynnon.com/) and unigenes with identity larger than 60% were selected.

### Comparative Expression Analysis

The R package DESeq was performed to identify differential gene expression [94]. This method represents the widely accepted and accurate analysis approaches of RNA-seq data. We first mapped high-quality reads to unigenes to calculate the number of reads mapped to each unigene in five samples. These raw read counts were then used as the input of DESeq to get the normalized signal for each unigene, and the fold change of unigene expression values with p-values compared to each other of the five samples was used to report differential expression. Those with p-value<0.05 were considered as significant differential expression. We performed cluster analysis of gene expression patterns with the Cluster [95], MeV [96] and Java treeview software packages [97].

## Supporting Information

Figure S1 **Length distribution of contigs, transcripts and unigenes.**
(TIF)

Figure S2 **Matching percentage of broccoli unigenes with different lengths to entries in public databases.**
(TIF)

Figure S3 **EggNOG classification of the broccoli seed and sprout transcriptome.**
(TIF)

Figure S4 **Classification of unigenes based on KEGG categorization.**
(TIF)

Table S1 **Characterization of raw data and trimmed data.**
(PDF)

Table S2 **Transcription factor members of every family detected in the broccoli seeds and sprouts.**
(PDF)
