# Correction: RNA-Seq Analysis of Transcriptome and Glucosinolate Metabolism in Seeds and Sprouts of Broccoli (*Brassica oleracea var. italic*)

**DOI:** 10.1371/journal.pone.0096880

**Published:** 2014-04-30

**Authors:** The PLOS ONE Staff

The word “*italic*” in the title of this article is incorrect and should be spelled “*italica*”. The correct title is: RNA-Seq Analysis of Transcriptome and Glucosinolate Metabolism in Seeds and Sprouts of Broccoli (*Brassica oleracea var. italica*).

The correct citation is: Gao J, Yu X, Ma F, Li J (2014) RNA-Seq Analysis of Transcriptome and Glucosinolate Metabolism in Seeds and Sprouts of Broccoli (*Brassica oleracea var. italica*). PLoS ONE 9(2): e88804. doi:10.1371/journal.pone.0088804
