# Supplementary figures and images for: RNA-Seq Analysis of Transcriptome and Glucosinolate Metabolism in Seeds and Sprouts of Broccoli (Brassica oleracea var. italic)
Source: PLoS One. 2014 Feb 27;9(2):e88804. doi: 10.1371/journal.pone.0088804 (PMC3937326; doi:10.1371/journal.pone.0088804)

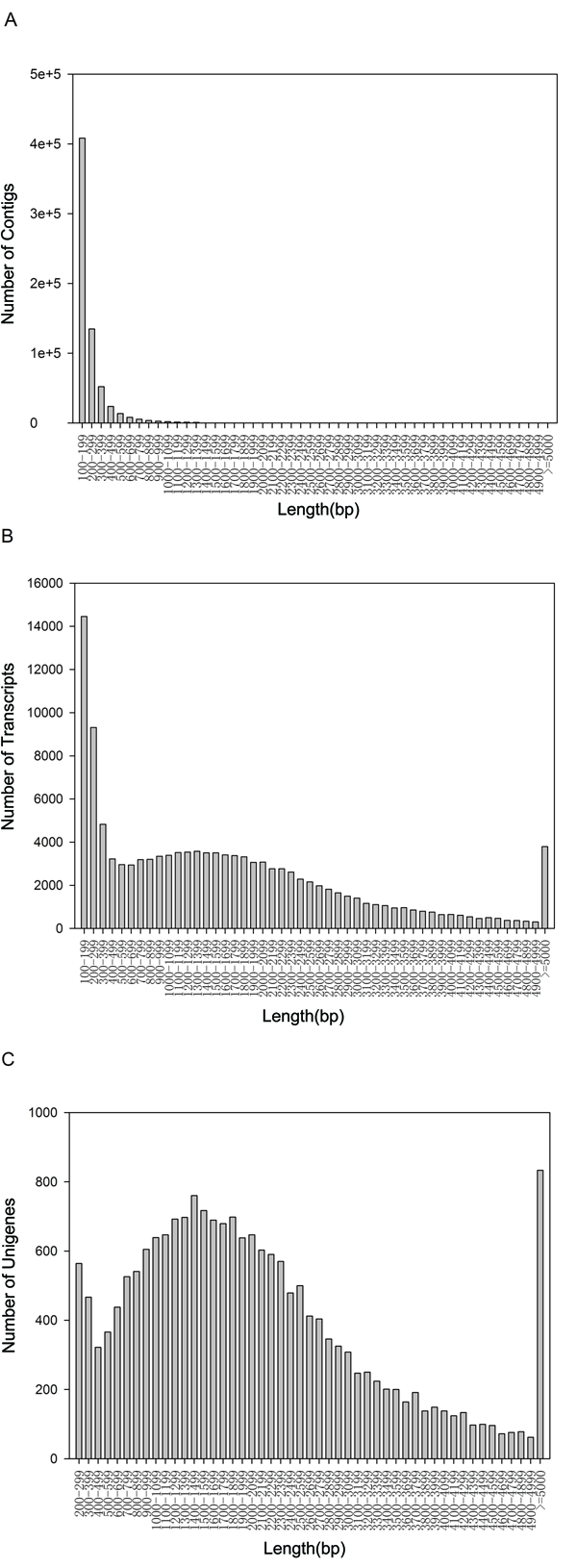

Supplement: Figure S1 — Length distribution of contigs, transcripts and unigenes. (TIF) [file pone.0088804.s001.tif]

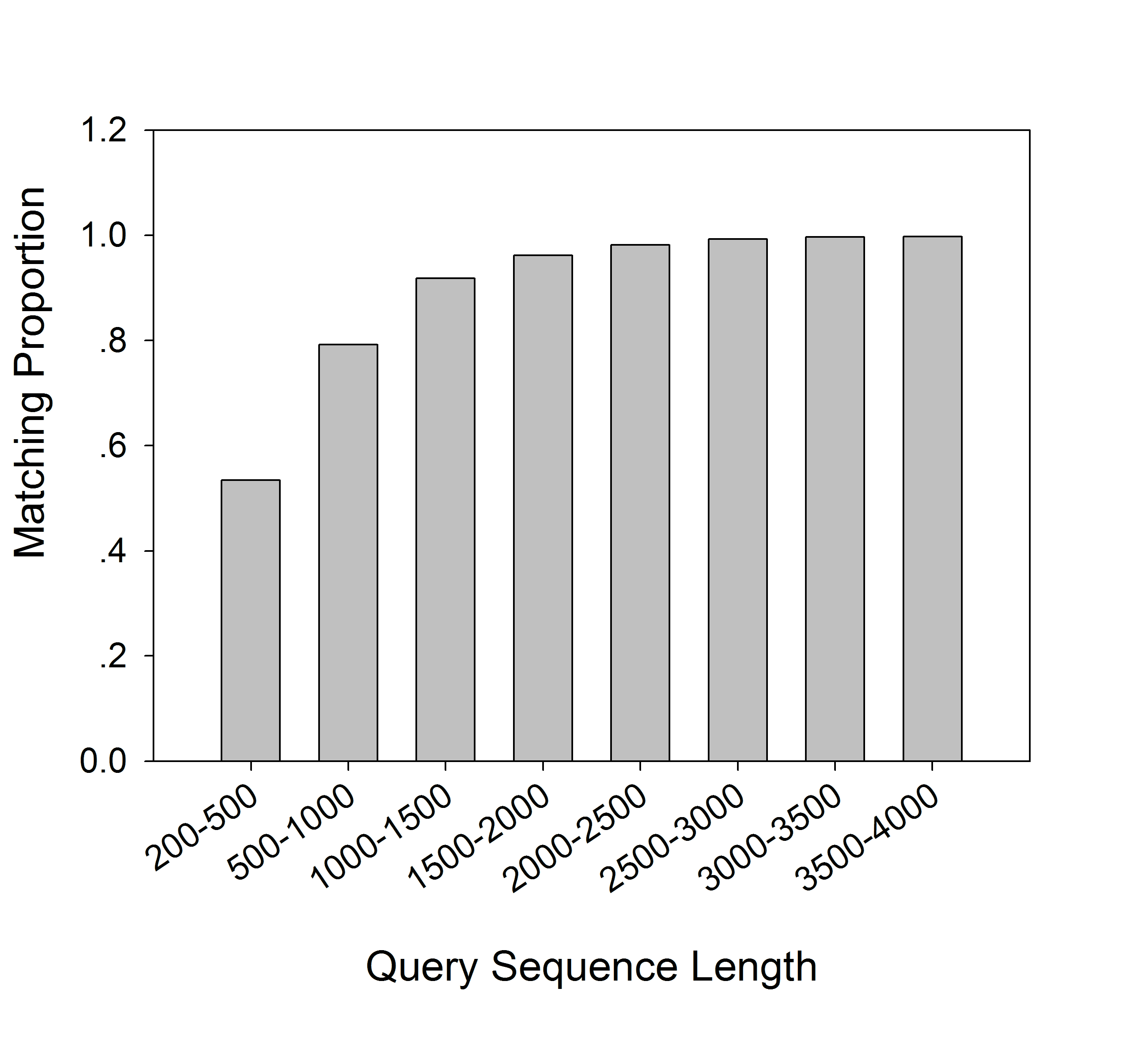

Supplement: Figure S2 — Matching percentage of broccoli unigenes with different lengths to entries in public databases. (TIF) [file pone.0088804.s002.tif]

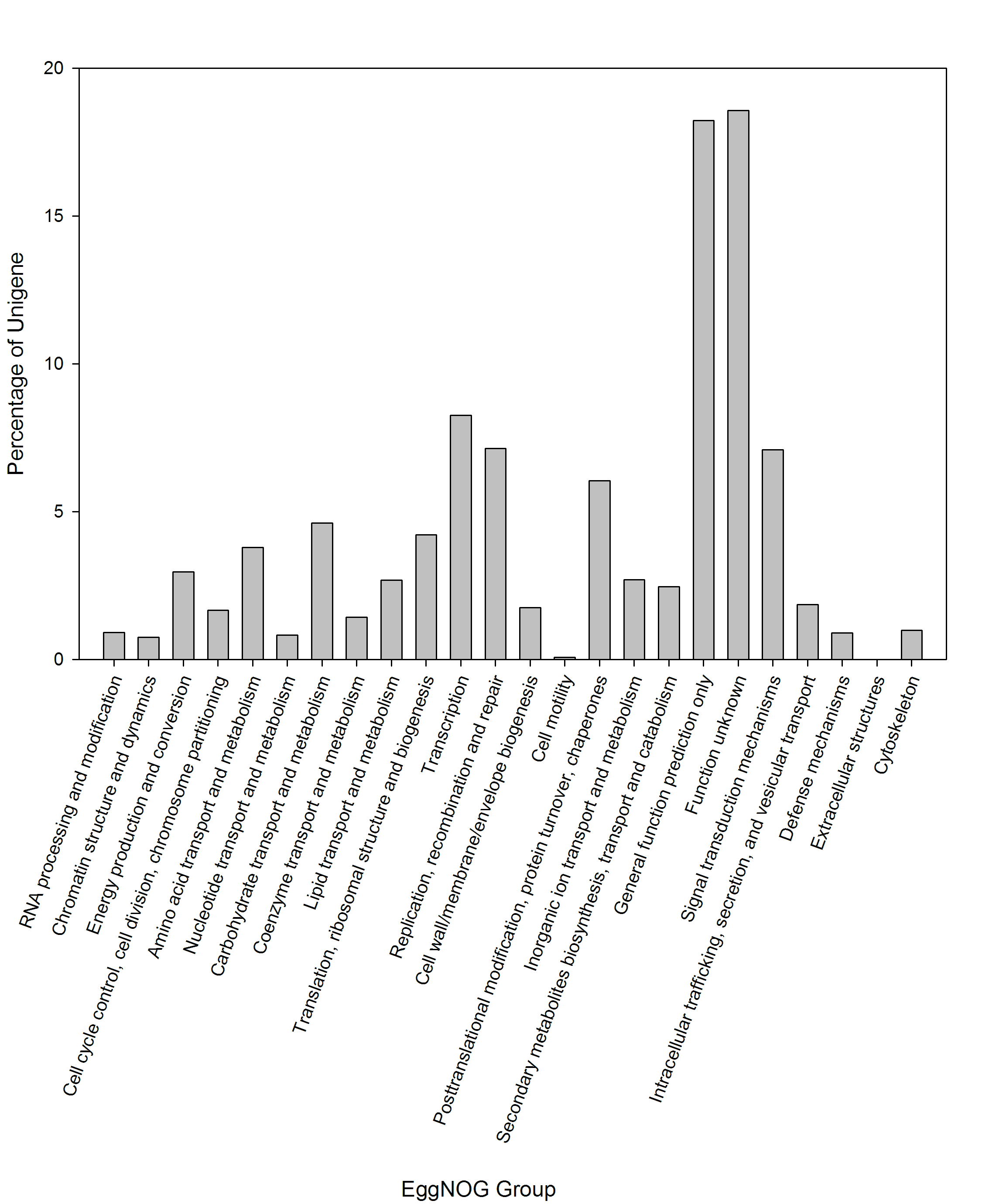

Supplement: Figure S3 — EggNOG classification of the broccoli seed and sprout transcriptome. (TIF) [file pone.0088804.s003.tif]

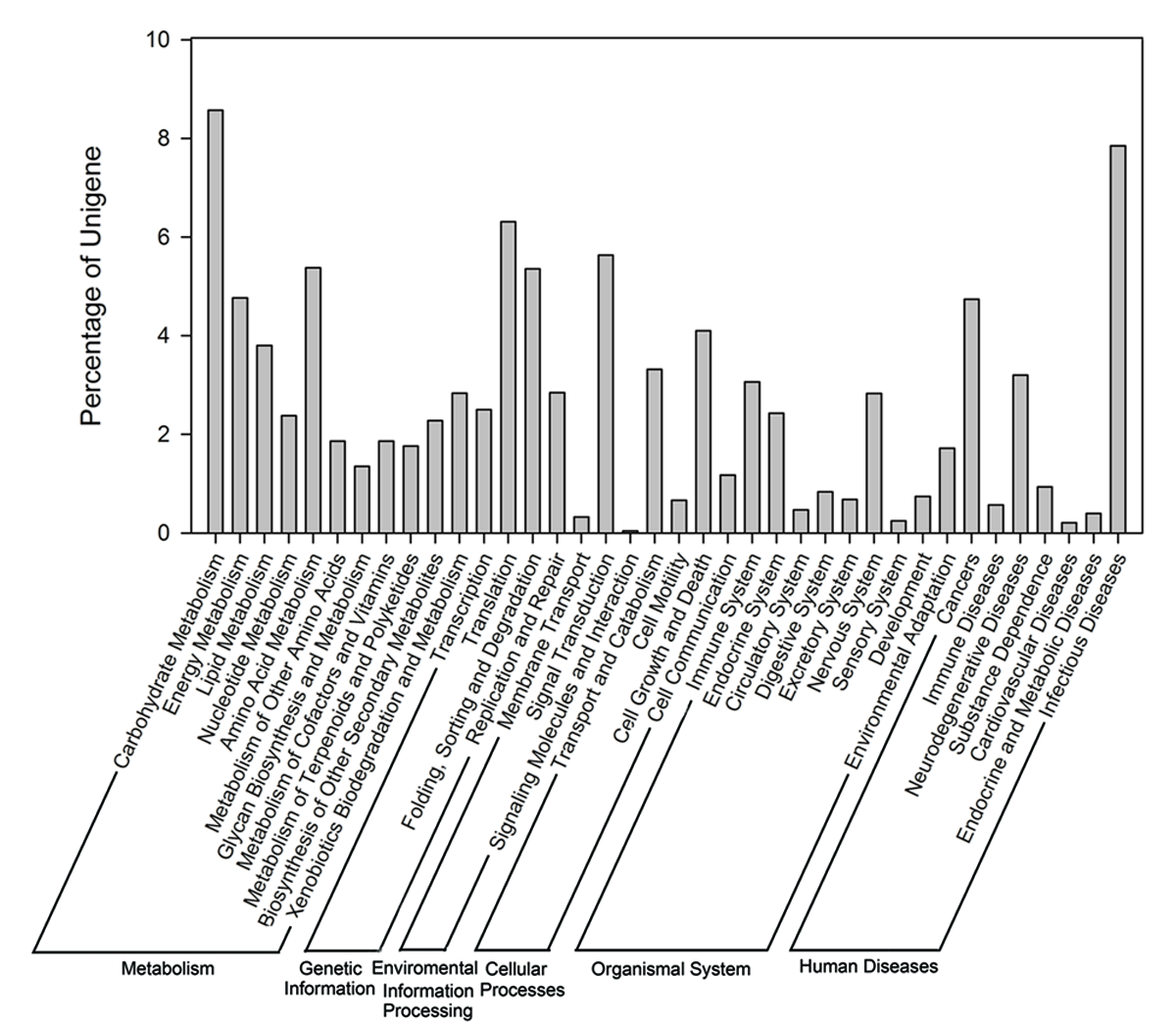

Supplement: Figure S4 — Classification of unigenes based on KEGG categorization. (TIF) [file pone.0088804.s004.tif]
