# Supplementary material for: RNA-Seq Analysis of Transcriptome and Glucosinolate Metabolism in Seeds and Sprouts of Broccoli (Brassica oleracea var. italic)
Source: PLoS One. 2014 Feb 27;9(2):e88804. doi: 10.1371/journal.pone.0088804 (PMC3937326; doi:10.1371/journal.pone.0088804)
Supplement: Table S1 — Characterization of raw data and trimmed data. (PDF) [file pone.0088804.s005.pdf]

Table S1. Characterization of raw data and trimmed data.

Sample	Reads	Raw Reads	Raw Data(bp)	Reads Len.(bp)
Seeds	R1	9754489	2448376739	251
	R2	9754489	2448376739	
	Paired	9754489	4896753478	
Cotyledon(3d)	R1	7452864	1870668864	251
	R2	7452864	1870668864	
	Paired	7452864	3741337728	
Cotyledon(7d)	R1	9205040	2310465040	251
	R2	9205040	2310465040	
	Paired	9205040	4620930080	
Cotyledon(11d)	R1	9891820	2482846820	251
	R2	9891820	2482846820	
	Paired	9891820	4965693640	
Euphylla(11d)	R1	6248763	1568439513	251
	R2	6248763	1568439513	
	Paired	6248763	3136879026	

Sample	Reads	Trimed Reads	Trimed Data(bp)	Useful Reads %	Useful Data %
Seeds	R1	8306466	1827922032	0.851553167	0.702920144
	R2	8306466	1614104627		
	Paired	8306466	3442026659		
Cotyledon(3d)	R1	6685661	1457313581	0.897059305	0.741961895
	R2	6685661	1318616449		
	Paired	6685661	2775930030		
Cotyledon(7d)	R1	8432659	1821956243	0.916091511	0.757531582
	R2	8432659	1678544233		
	Paired	8432659	3500500476		
Cotyledon(11d)	R1	8492069	1864708802	0.858494089	0.707426511
	R2	8492069	1648154524		
	Paired	8492069	3512863326		
Euphylla(11d)	R1	5746413	1239294058	0.919608089	0.760055501
	R2	5746413	1144908103		
	Paired	5746413	2384202161		
