# Supplementary material for: RNA-Seq Analysis of Transcriptome and Glucosinolate Metabolism in Seeds and Sprouts of Broccoli (Brassica oleracea var. italic)
Source: PLoS One. 2014 Feb 27;9(2):e88804. doi: 10.1371/journal.pone.0088804 (PMC3937326; doi:10.1371/journal.pone.0088804)
Supplement: Table S2 — Transcription factor members of every family detected in the broccoli seeds and sprouts. (PDF) [file pone.0088804.s006.pdf]

Table S2. Transcription factor members of every family detected in the broccoli seeds and sprouts

TF Family	Members	TF Family	Members
AP2-EREBP	109	C2C2-CO-like	12
bHLH	99	GRF	11
MYB	84	BES1	11
HB	77	HMG	10
C3H	69	TUB	9
NAC	67	RWP-RK	9
C2H2	65	Alfin-like	9
bZIP	61	Sigma70-like	7
Orphans	59	PLATZ	7
MYB-related	56	MBF1	7
WRKY	54	E2F-DP	7
PHD	49	CAMTA	7
SNF2	37	ARR-B	7
CCAAT	37	ARID	7
G2-like	33	TAZ	6
SET	30	SRS	6
GNAT	30	CPP	6
C2C2-GATA	30	C2C2-YABBY	6
C2C2-Dof	30	BBR/BPC	6
GRAS	27	SWI/SNF-SWI3	5
AUX/IAA	26	Pseudo_ARR-B	5
Trihelix	24	LIM	5
MADS	23	EIL	5
mTERF	21	DDT	5
HSF	21	DBP	5
TRAF	20	GeBP	4
ABI3VP1	20	VOZ	3
TCP	18	PBF-2-like	3
LOB	16	ULT	2
Jumonji	15	Rcd1-like	2
FHA	15	RB	2
zf-HD	14	MED7	2
SWI/SNF-BAF60b	14	LUG	2
ARF	14	SOH1	1
Tify	13	LFY	1
BSD	13	IWS1	1
SBP	12	HRT	1
OFP	12	CSD	1
FAR1	12	Coactivator_p15	1
